# First Episode Psychosis in Patients Aged 18 to 30 Admitted Involuntarily: Characteristics and Risk Factors for Functional Non-Remission

**DOI:** 10.3390/brainsci15070697

**Published:** 2025-06-28

**Authors:** Maria El Helou, Matthieu Hein, Beni-Champion Cimpaye, Benjamin Wacquier, Anaïs Mungo

**Affiliations:** 1 Faculté de Médecine, Université Libre de Bruxelles (ULB), 1070 Brussels, Belgium; maria.el.helou@ulb.be (M.E.H.); benichampion.cimpaye@ulb.be (B.-C.C.); 2 Laboratoire de Psychologie Médicale et Addictologie (ULB312), CHU Brugmann, Université Libre de Bruxelles (ULB), 1020 Brussels, Belgium; 3 Hôpital Universitaire de Bruxelles—Site Anderlecht, Service de Psychiatrie et Laboratoire du Sommeil, Université Libre de Bruxelles (ULB), 1070 Brussels, Belgium; benjamin.wacquier@hubruxelles.be; 4 Centre Hospitalier Le Domaine-ULB, Service de Psychiatrie, Université Libre de Bruxelles (ULB), 1420 Braine l’Alleud, Belgium; anais.mungo@ulb.be

**Keywords:** first-episode psychosis, early intervention, duration of untreated psychosis, involuntary hospitalization, functional remission, risk factors

## Abstract

Introduction: This study aimed to explore the clinical and psychosocial characteristics associated with functional non-remission in young adults involuntarily hospitalized for a first episode of psychosis (FEP), focusing on the role of duration of untreated psychosis (DUP) and contextual vulnerabilities. Material and method: We conducted a retrospective monocentric study including 123 patients aged 18–30 who were involuntarily admitted between 2013 and 2023 for a first psychotic episode. Sociodemographic, clinical, and care-related data were extracted from medical records. Functional remission was defined as a Global Assessment of Functioning (GAF) score ≥70 at discharge. Univariate and multivariate logistic regressions were used to identify predictors of functional non-remission. Results: Only 48.8% of patients achieved functional remission at discharge. Social isolation, low socioeconomic status, unemployment, lack of structured activities, and a DUP ≥ 4 weeks were significantly associated with functional non-remission. After multivariate logistic regressions, DUP ≥ 4 weeks remained an independent predictor of functional non-remission. Conclusions: Involuntary admission per se was not a direct predictor of poor outcome. Our findings highlight the critical role of prolonged DUP and psychosocial vulnerability in the trajectory of early psychosis. Early detection strategies, psychosocial support integration, and individualized care planning are essential to improve outcomes among young people experiencing FEP under compulsory admission.

## 1. Introduction

Psychotic disorders (PD) are severe and prevalent mental health conditions, affecting approximately 3% of the population over the course of their lifetime. They encompass a range of symptoms indicative of a loss of contact with reality, such as hallucinations, delusions, disorganized speech, and other cognitive disturbances [1]. Schizophrenia constitutes only a subset of psychotic disorders, which also include organic psychoses (e.g., delirium, dementia, brain lesions) and affective disorders with psychotic features such as mania or psychotic depression [2].

The first episode of psychosis (FEP) represents a critical period in the trajectory of psychotic disorders, with significant implications for both clinical and functional prognosis. FEPs typically emerges during adolescence or early adulthood—a developmental phase characterized by heightened vulnerability and major psychosocial transitions [3]. Early and appropriate intervention is essential to mitigate the often-traumatic experience associated with psychosis, including the disclosure of the diagnosis, hospitalization, and treatment procedures. FEP can considerably disrupt daily functioning, social relationships, and academic or occupational performance. Timely intervention has been shown to influence long-term outcomes, notably by reducing the risk of chronicity and treatment resistance [2,4]. However, recovery trajectories following FEP are highly heterogeneous: some individuals show rapid improvement, while others exhibit persistent treatment resistance. Long-term incomplete remission is reported in 10% to 50% of cases [5]. This lack of symptomatic and/or functional remission represents a major public health concern, as it is associated with increased clinical deterioration, repeated hospitalizations, chronicity, neurotoxic effects of relapse, poor quality of life, and impaired global functioning [6].

Involuntary hospitalization is frequently employed in psychiatric care, particularly when patients present a significant risk to themselves or others. It is often regarded as a marker of clinical severity in FEP and is commonly associated with acute symptomatology, significant functional impairment, and poor insight. Several studies have demonstrated that clinical, sociodemographic, and environmental factors may influence the course of FEP and the likelihood of achieving full remission [7]. Nevertheless, a substantial proportion of patients fail to attain symptomatic and functional remission, which may compromise their social and occupational reintegration [8]. This admission modality raises important questions regarding its impact on clinical evolution and the risk factors associated with functional non-remission.

### 1.1. Impact of Duration of Untreated Psychosis (DUP)

The duration of untreated psychosis (DUP) is a critical determinant of clinical outcomes in FEP. A recent meta-analysis confirmed that prolonged DUP is associated with more severe symptomatology, diminished treatment response, and an increased risk of relapse [9]. More specifically, the study demonstrated a significant correlation between extended DUP and the exacerbation of both positive and negative symptoms, as well as reduced chances of achieving symptomatic remission [9].

### 1.2. Characteristics of Involuntarily Hospitalized Patients

Patients admitted under compulsory care often present distinct clinical profiles. A study by Georgaca et al. (2023) identified three subtypes among a hospitalized population: one characterized by disorganized psychotic symptoms associated with impaired global functioning and poor treatment adherence; a second typified by active positive symptoms in younger patients with relatively preserved functioning; and a third dominated by depressive symptomatology, more commonly associated with voluntary hospitalization [10]. The first two profiles were significantly linked to involuntary admission, suggesting more severe clinical presentation and lower treatment engagement prior to hospitalization [10].

Furthermore, a cohort study conducted in Melbourne reported that migration status, aggressive behavior, and more severe psychotic symptoms increased the likelihood of involuntary admission for FEP [11]. Interestingly, and contrary to expectations, longer DUP was associated with lower rates of hospitalization, likely due to a progressive chronicity that diminished clinical urgency [11].

### 1.3. Effects of Involuntary Hospitalization on Prognosis

The impact of involuntary hospitalization on patient outcomes remains a matter of debate. Some studies suggest it may be beneficial by providing a structured environment and rapid access to care, thereby facilitating clinical stabilization [11,12]. However, other research emphasizes potential adverse effects, such as a deteriorated therapeutic alliance, decreased post-discharge adherence, and an increased risk of rehospitalization [12].

Longitudinal analyses of involuntarily hospitalized patients reveal substantial heterogeneity in clinical trajectories. A 10-year follow-up study identified five distinct symptomatic pathways, ranging from full remission to chronic symptom persistence [8]. Moreover, a five-year follow-up of the Danish OPUS trial found that patients receiving specialized care experienced superior symptomatic and functional outcomes compared to those receiving standard treatment [7]. These findings underscore the importance of individualized intervention strategies tailored to patient profiles.

### 1.4. Risk Factors for Functional Non-Remission

In recent years, research has increasingly focused on identifying patient-related predictors of functional non-remission. Key risk factors include low premorbid functioning and poor insight [13,14,15]. Younger age at onset, substance abuse, history of trauma, and low educational attainment have also been significantly associated with poorer outcomes [16,17]. Additionally, the presence of psychiatric comorbidities at onset, such as negative symptoms [18] or thought disorganization [14], may predict poor response. Treatment-related factors such as poor adherence [19], lack of early treatment response [15], and, notably, prolonged DUP [6,20] have also been implicated.

Beyond DUP, a meta-analysis by Santesteban-Echarri et al. (2017) identified poor social support, substance use, and low premorbid functioning as additional predictors of unfavorable trajectories [21]. DUP remains one of the most robust predictors of functional non-remission [9,20,21]. Psychotic symptoms may increase psychological stress and exert neurotoxic effects on the brain. This interaction could contribute to prolonged and complex illness courses, impairing treatment efficacy and the likelihood of functional remission [22,23].

These findings underscore the critical need for early and appropriate intervention to optimize clinical and functional outcomes.

Recent studies have also emphasized the role of specialized services for youth at high clinical risk, such as those with an At-Risk Mental State (ARMS). Patients receiving early intervention through these programs exhibit reduced hospitalization rates and improved functional remission outcomes compared to those entering FEP care services directly [24].

### 1.5. Study Objective

The aim of the present study is to analyze the clinical and sociodemographic characteristics of young adults aged 18 to 30 years who are involuntarily hospitalized for a first episode of psychosis, and to identify risk factors associated with functional non-remission. Drawing upon empirical data and epidemiological models, the study will explore the influence of DUP, sociodemographic variables, and treatment modalities on clinical outcomes. A better understanding of these factors could inform the development of optimized early intervention strategies and ultimately improve patient prognosis.

## 2. Materials and Methods

### 2.1. Study Design

This study is a retrospective, monocentric investigation conducted in the Adult Psychiatry Department of Erasme Hospital.

### 2.2. Population

Participants were retrospectively identified based on data available in their medical records. The inclusion and exclusion criteria for this study are available in Table 1. Based on these criteria, 123 involuntarily hospitalized patients aged 18 to 30 years presenting with FEP were included in the study.

### 2.3. Methodology

Sociodemographic variables collected included sex, age (in years), social isolation, socioeconomic level of the residential municipality, migration history, education level, employment status, and engagement in leisure or sports activities.

Clinical history comprised previous psychiatric follow-up, familial difficulties, previous psychiatric hospitalizations, history of suicide attempts, family history of psychiatric disorders, and prior traumatic events. In this study, family difficulties included issues of family involvement in the adequate care of patients.

Initial clinical assessment included duration of psychotic symptoms prior to hospitalization (in weeks), psychotropic medication at admission, presence of somatic comorbidities, cannabis use disorder, other substance use disorders, number of psychiatric comorbidities, and Global Assessment of Functioning (GAF) score at admission. Regarding cannabis use disorder and other substance use disorders, they were coded binary (absent or present) because the limited size of our sample did not allow a more detailed analysis of the different consumptions.

Care-related variables included duration of hospitalization, confirmation of involuntary hospitalization, prolongation of the involuntary stay beyond 40 days, administration of long-acting injectable antipsychotics, discharge diagnosis, GAF score at discharge, and presence of treatment response or remission. In this study, since patients were required to take their medication under the supervision of the unit team, no measurement of treatment adherence was carried out given this context of compulsory care. Furthermore, given the absence of specific measurement, the speed of response to psychotic treatment was indirectly assessed by the length of hospitalization. Indeed, given the medico-legal context associated with the hospitalizations of the patients included in this study, a short duration of hospitalization is generally associated with a faster response to psychotic treatments allowing the end of the obligation for patients to remain hospitalized.

DUP was retrospectively estimated from medical records based on clinical data collected during semi-structured interviews conducted upon admission of patients to the unit. In addition, during these semi-structured interviews with patients, heteroanamneses of relatives were systematically carried out in order to allow the best possible reconstruction of the timeline of the occurrence of initial psychotic symptoms (hallucinations, delusional ideas, disorganization) as well as the date of the potential initiation of antipsychotic treatment. Thus, for this study, DUP was only assessed based on a contextualized clinical assessment without the use of standardized measurement scales.

In this study, functional remission was defined as the absence of significant symptoms impairing functioning at discharge (GAF ≥ 70) [25,26], while treatment response was defined as a clinical improvement with GAF ≥ 50.

### 2.4. Statistical Analyses

Statistical analyses were performed using Stata version 14. Normality of distribution was assessed using histograms, box plots, and quantile–quantile plots. Homogeneity of variance was tested using Levene’s test.

The sample was divided into two groups: patients who achieved functional remission and those who did not. Descriptive analyses were performed using percentages and absolute numbers for categorical variables, and median and interquartile range (P25–P75) for continuous variables.

Due to non-normal distribution of most continuous variables, comparisons were conducted using Wilcoxon rank-sum tests. Categorical data were analyzed using Chi-square tests.

Univariate logistic regression models were used to assess the association between risk of functional non-remission and duration of psychotic symptoms before hospitalization (categorized as <4 weeks vs. ≥4 weeks), as well as potential confounders. Subsequently, multivariate logistic regression was performed adjusting for confounding factors that were statistically significant in univariate analyses. In our study, we chose a cut-off for the duration of psychotic symptoms before hospitalization of 4 weeks because it was the duration of psychotic symptoms before hospitalization with the best sensitivity and specificity for the prediction of functional non-remission in our sample.

Model fit was evaluated using the Hosmer–Lemeshow test, and model specification was verified using the Link test. Moreover, the absence of multicollinearity in the final model was verified by the variance inflation factor method.

Following the conditions of use of multivariate logistic regression analyses (number of subjects per cofactor ≥ 10) [27,28], each of the two groups of patients for this study had to contain at least 60 subjects (10 subjects × 6 cofactors) to ensure the validity of the analyses performed, which was achieved in this study.

Thus, for this study, despite the limited sample size and the relatively high number of factors studied, no problems arose for the selection of variables in the final multivariate model given the strict respect of the conditions of use of the analyses performed (absence of multicollinearity between variables and sufficient number of subjects per cofactor in each group).

A *p*-value < 0.05 was considered statistically significant.

## 3. Results

### 3.1. General Characteristics of the Sample

The analyzed sample comprised 123 patients involuntarily hospitalized for a first episode of psychosis. Of these, 47 were women (38.2%) and 76 were men (61.8%). The median age was 24 years, with 56.1% of patients being younger than 25. Regarding educational level, 23.6% had completed higher education, 26.8% vocational secondary education, 18.7% general secondary education, and 30.9% had not completed secondary school.

More than half of the patients (55.3%) had a cannabis use disorder, and 23.6% had another substance use disorder. The median duration of untreated psychotic symptoms prior to admission was 2 weeks. The median GAF score at admission was 35, indicating significant global functional impairment, and 65 at discharge.

Regarding DUP, the median was, respectively, 2 weeks (95% confidence interval: 1.00–2.87), 1 week (95% confidence interval: 1.00–2.00), and 3 weeks (95% confidence interval: 1.00–4.00) for the whole sample, for patients with functional remission, and for patients without functional remission.

Detailed results are available in Table 2, Table 3 and Table 4.

### 3.2. Inpatient Care

The median length of hospitalization was 6 weeks. Involuntary hospitalization was maintained for 40 days in 75 patients (61%). A prolonged involuntary hospitalization (beyond 40 days) was implemented for 35 patients (28.5%). A long-acting injectable antipsychotic was prescribed to 32 patients (26%). Treatment response was observed in 104 patients (84.6%), while functional remission was achieved in 60 patients (48.8%).

Detailed results are available in Table 4.

### 3.3. Comparison Between Functional Remission and Functional Non-Remission Groups

Among the 123 patients, 60 (48.8%) achieved the criteria for functional remission, defined as a GAF score ≥ 70 at discharge. Conversely, 63 patients (51.2%) did not meet functional remission criteria.

No significant differences were observed between the two groups regarding age, sex, education level, migration history, personal or familial psychiatric history, psychotropic treatment prior to hospitalization, presence of somatic comorbidities, substance use, confirmation or prolongation of involuntary admission, or discharge diagnoses.

However, several psychosocial and clinical variables were significantly associated with functional non-remission: social isolation (*p* = 0.005), low socioeconomic status (*p* = 0.011), unemployment (*p* = 0.028), and lack of engagement in leisure or sports activities (*p* = 0.009) were more prevalent in the functional non-remission group. Clinically, a duration of psychotic symptoms ≥ 4 weeks before hospitalization (*p* = 0.001), a GAF score < 30 at admission (*p* = 0.001), and hospitalization lasting more than 8 weeks (*p* = 0.002) were significantly associated with absence of functional remission.

Detailed results are available in Table 2, Table 3 and Table 4.

### 3.4. Univariate Regression Analysis

Social isolation, low socioeconomic status, unemployment, absence of leisure or sports activities, a GAF score < 30 at admission, and hospitalization duration > 8 weeks were all significantly associated with functional non-remission in this cohort of young adults aged 18 to 30 years.

A DUP ≥ 4 weeks prior to hospitalization increased the risk of functional non-remission by 3.5 times (OR = 3.50; 95% CI: 1.59–7.70; *p* = 0.002).

Detailed results are available in Table 2, Table 3 and Table 4.

### 3.5. Multivariate Regression Analysis

The aim of the multivariate analysis was to determine whether the duration of psychotic symptoms prior to involuntary hospitalization (<4 weeks vs. ≥4 weeks) was an independent predictor of functional non-remission, after adjusting for potential confounders.

After adjusting for social isolation, socioeconomic status, employment, and engagement in leisure or sports activities (Model 1), a DUP ≥ 4 weeks remained significantly associated with functional non-remission (adjusted OR = 3.11; 95% CI: 1.32–7.33; *p* = 0.009).

**Figure 1:**
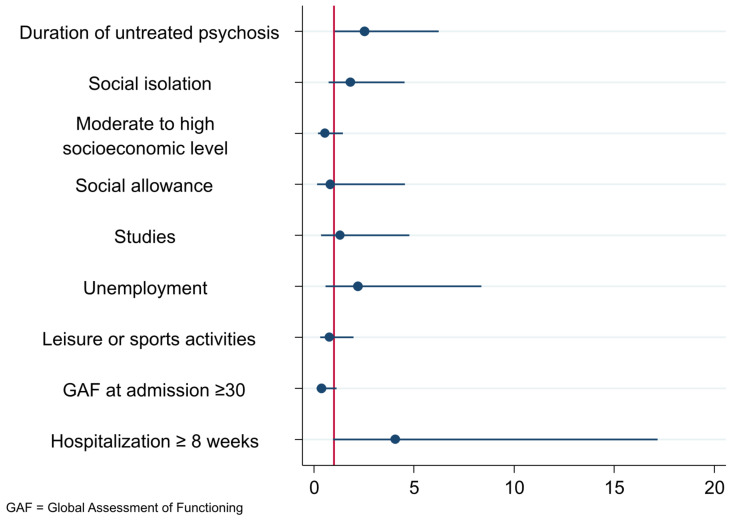
Forest plot of adjusted OR for final multivariate model.

Further adjustment for GAF score at admission and duration of hospitalization (Model 2) slightly attenuated the effect but did not eliminate the significant association (adjusted OR = 2.54; 95% CI: 1.03–6.24; *p* = 0.043). The forest plot of adjusted OR for this final multivariate model is available in Figure 1.

Overall, multivariate logistic regression analysis confirmed that a DUP longer than 4 weeks prior to involuntary hospitalization was independently associated with a higher risk of functional non-remission among involuntarily hospitalized FEP patients aged 18 to 30 years.

Detailed results are available in Table 5.

## 4. Discussion

### 4.1. Interpretation of Main Findings

In our study, the median age of patients experiencing a FEP was 24 years, which aligns with the age range typically observed for the onset of psychotic decompensation, usually between 18 and 25 years [29].

Our findings also highlight several key results regarding young adults involuntarily hospitalized for FEP. The overall functional remission rate observed (48.8%) is lower than those reported in the literature [30,31,32]. This discrepancy may be attributed to the increased clinical severity—more pronounced symptomatology and greater functional impairment—observed in involuntarily hospitalized patients. Prior studies often include a broader range of FEP presentations, including less severe cases.

Remission at discharge, although indicative of a favorable short-term response, does not reliably predict the long-term symptomatic and functional course. Indeed, several longitudinal studies conducted among patients with a FEP have demonstrated considerable heterogeneity in clinical trajectories. For instance, Verma et al. (2012) found that while more than half of the patients achieved symptomatic remission at two years (54.1%), 58.4% experienced functional remission and only 29.4% met both symptomatic and functional remission criteria simultaneously [32]. In the OPUS cohort, five-year follow-up results showed that only 18% of patients fulfilled recovery criteria, whereas 13% remained institutionalized [7]. At ten years, Austin et al. (2015) identified four distinct symptomatic trajectories, highlighting the frequent persistence of negative symptoms and long-term clinical fluctuations [8]. These findings confirm that clinical remission in the acute phase cannot be considered a sufficient indicator of sustained recovery and underscores the need for longitudinal studies to better understand the determinants of favorable outcomes in this population. However, before focusing on this long-term evolution, obtaining functional remission after FEP must remain the first step to be achieved in the recovery journey of patients [33].

Patients in our sample demonstrated a profile marked by psychosocial vulnerability (e.g., social isolation, unemployment, lack of structured activity) and significant baseline dysfunction. The median duration of psychotic symptoms prior to hospitalization was 2 weeks in our sample, contrasting with the global average of 43 weeks and a median of 14 weeks reported in the literature. In Europe, the median DUP is around 12 weeks [34]. This discrepancy may reflect the acute nature of episodes leading to involuntary admission, indicative of an abrupt onset of psychosis and, consequently, a shorter untreated period.

#### Predictive Factors for Functional Non-Remission

Among the identified predictors of functional non-remission, DUP emerged as a robust and independent factor. A DUP greater than four weeks was significantly associated with an increased risk of functional non-remission in involuntarily hospitalized young adults with FEP. This association remained statistically significant even after adjustment for various clinical and sociodemographic variables, as evidenced by the multivariate regression model (adjusted OR = 2.54; 95% CI = 1.03–6.24; *p* = 0.043). These findings are consistent with existing literature indicating that prolonged DUP is among the strongest predictors of poor prognosis in early psychotic disorders [9,20,35].

The underlying mechanism may involve the potential neurotoxic effects of untreated psychosis on brain structures [22]. Several hypotheses have been proposed: prolonged hyperdopaminergia may result in reduced brain volume, altered neural connectivity, and impaired neuroplasticity, particularly under chronic stress and glucocorticoid exposure [23,30,36]. However, this neurotoxicity hypothesis remains debated, and causality cannot be firmly established. Several neuroimaging studies have reported associations between longer DUP and subtle brain structural changes. These include reduced gray matter volume in the superior temporal gyrus, temporal, occipital, and fusiform cortices, caudate nucleus, limbic areas, and hippocampus, as well as the inferior-orbital and parietal regions [37,38,39,40,41]. While these findings suggest a potential detrimental effect of prolonged untreated psychosis, most studies were limited by retrospective DUP assessment, prior antipsychotic exposure, or small sample sizes. Some authors have also argued that longer DUP may reflect underlying illness heterogeneity rather than a direct neurotoxic process. Therefore, the interpretation of DUP as a causative factor in brain changes should remain cautious, and further longitudinal neuroimaging studies are needed to clarify this relationship. A longer DUP may also expose individuals to prolonged psychosocial stress or traumatic experiences, which could hinder psychological development, induce neurobiological changes, and compromise emotional regulation, thereby reducing the likelihood of remission [42]. Furthermore, prolonged DUP has been associated with social isolation, poor access to care, and disruptions in socio-occupational functioning—all factors known to worsen clinical outcomes [21,43].

Our findings also emphasize the relevance of contextual and social factors in FEP trajectories. Social isolation (OR = 2.88; *p* = 0.005), low socioeconomic status (OR = 0.36; *p* = 0.012), lack of leisure activities (OR = 0.38; *p* = 0.010), and unemployment (OR = 4.33; *p* = 0.033) were all significantly associated with functional non-remission. These results are in line with prior studies that highlight the importance of good premorbid adjustment and a supportive social environment as favorable prognostic factors in FEP [13,14,15,44]. In contrast, the predictors of functional non-remission in our sample suggest poor premorbid adjustment, possibly reflecting early difficulties with stress regulation, interpersonal relationships, and social adaptation.

These vulnerabilities may impair the effectiveness of therapeutic interventions, by hindering treatment adherence and exacerbating core symptomatology. Although factors such as trauma history, low educational attainment, and substance use are also frequently associated with poorer treatment response in the literature, these associations were not observed in our sample—likely due to the relatively small sample size [17,19]. These results underscore the need for comprehensive care models that incorporate both pharmacological treatment and psychosocial rehabilitation efforts, including community-based interventions.

Hospitalization duration also appears to be a marker of clinical severity. In our study, a hospital stay exceeding 8 weeks was strongly associated with functional non-remission (OR = 6.47; *p* = 0.005), likely reflecting both delayed response to treatment and more severe clinical presentations at admission. Indeed, patients in the functional non-remission group had significantly lower GAF scores at admission (median = 30 vs. 40; *p* = 0.003) and discharge (median = 60 vs. 75; *p* < 0.001), confirming their more impaired functional status. This aligns with findings from Kinon et al. (2008) [45], who noted that early treatment response in FEP is a strong predictor of later remission.

### 4.2. Involuntary Hospitalization: Marker of Severity Rather than Causal Factor?

Although central to our study, involuntary hospitalization did not emerge as a direct predictor of functional non-remission. This finding suggests that this admission modality is more likely to reflect acute symptomatic severity rather than being intrinsically deleterious [11]. Nevertheless, prior studies have highlighted potential long-term consequences of coercive care, including impaired therapeutic alliance and reduced post-discharge adherence [12].

### 4.3. Therapeutic Perspectives

Our findings strongly support the development of specialized early intervention services, such as At-Risk Mental State (ARMS) programs, which aim to identify and treat individuals before the onset of full-blown psychosis [3]. These early intervention models are characterized by continuity of care and dimensional, personalized approaches [35]. Such programs have demonstrated their efficacy in reducing DUP, lowering rates of involuntary hospitalization, and improving clinical outcomes [3,24].

However, tailoring interventions for patients at high risk of functional non-remission remains insufficiently defined in the literature. Key priorities should include early assessment, prompt initiation of appropriate treatment, involvement of family members, access to psychotherapy, and strong support for social and occupational reintegration [46,47].

A swift introduction of second-generation antipsychotics at moderate and regularly adjusted doses is generally recommended. In the presence of functional non-remission risk factors—particularly prolonged DUP—treatment protocols may need to be adapted. This may include the early use of clozapine, traditionally reserved as a second-line treatment, which may promote better outcomes if introduced sooner [47]. Family engagement is also crucial for enhancing treatment adherence and preventing relapse [46].

### 4.4. Limitations

This study presents several methodological limitations. Its retrospective nature may introduce biases related to data collection and interpretation, particularly due to the variable quality of medical records. In addition, being a monocentric study limits the generalizability of the findings. Although the study population was homogeneous in terms of age and diagnosis, the relatively small sample size (*n* = 123) further restricts generalizability. Finally, restricting the study to participants aged 18 to 30 excludes younger individuals who may also experience FEP.

The focus on involuntarily hospitalized patients may overrepresent more severe cases, limiting comparability with broader FEP cohorts. Indeed, a selection bias could exist due to the fact that only involuntarily hospitalized patients were included in this study. This restriction does not stem from a methodological choice but from a structural constraint, as the unit in question only admits such patients. As a result, there is a limitation in the generalizability of the findings, which cannot be extrapolated to populations admitted voluntarily since they are generally characterized by a better clinical profile (lower symptom severity, better treatment adherence, or lower psychosocial vulnerability) than those admitted involuntarily. Thus, given the existence of these limitations to generalize our results to patients admitted voluntarily with FEP, it seems necessary not to limit the study of functional non-remission factors only to involuntarily hospitalized patients but to also extend future investigations to those hospitalized voluntarily to identify factors specific to this particular subpopulation.

An important limitation of our study lies in the absence of follow-up after hospital discharge. As such, the observed functional remission is assessed only at a single time point at the end of the acute phase, without any information on its long-term stability. However, longitudinal studies have shown that the course of psychosis may vary significantly over the years following a first episode [8,32]. Long-term follow-up studies are therefore necessary to better characterize the clinical trajectory of these patients beyond the initial hospitalization.

Moreover, a methodological limitation lies in the exclusive use of the GAF scale, which does not allow for a specific assessment of psychotic symptoms and cannot substitute for a structured symptom evaluation (e.g., using the PANSS or BPRS). However, some studies have used the GAF as a criterion for functional remission, particularly in contexts where detailed symptomatic data are not available [32,48]. In line with this approach, we chose to define our outcome as functional remission, rather than full clinical remission. Future studies should incorporate more specific clinical tools to allow for a more comprehensive assessment of remission.

Furthermore, the definition of functional remission used in this study is based on a GAF score ≥ 70. Although this threshold may appear strict, it was chosen to reflect a high level of functioning, compatible with social, occupational, or academic autonomy, without major impairments [25,26]. The GAF provides a global and synthetic measure of functioning, encompassing psychological, social, and occupational dimensions. Its use is justified by its ease of administration, clinical relevance in naturalistic contexts, and its validity as a recognized proxy for quality of life, treatment adherence, risk of rehospitalization, and social and occupational integration.

Several previous studies have also used the GAF as a primary or complementary indicator to define functional remission, with thresholds ranging from ≥60 to ≥65 [32,48,49,50]. In our case, the absence of symptom rating scales in the retrospective dataset led us to rely on the GAF as the sole criterion. However, to avoid a potential overestimation of functional remission secondary to this exclusive use of GAF, we decided to use a stricter score on this scale (≥70) in this study rather than the other cut-offs usually used (≥60 or ≥65) [32,48,49,50]. Nevertheless, one potential risk of using this stricter cut-off for GAF score (≥70) may be an underestimation of remission in some patients with residual but manageable symptoms. Thus, in order to accurately reflect the scope of our evaluation, we therefore chose to refer to functional remission rather than clinical remission, while also acknowledging the limitations of this approach and emphasizing the need for future prospective studies to include specific symptom rating scales such as the PANSS or the BPRS.

In addition, the measurement of DUP constitutes a limitation of our study. It was determined based on a semi-structured interview conducted at admission, which included a heteroanamnestic assessment with the patient’s relatives, allowing for a certain degree of reliability in reconstructing the chronology. Nevertheless, the absence of a standardized scale and the memory biases inherent to retrospective data collection may affect the accuracy of this estimation. This variability should be taken into account when interpreting the associations observed between DUP and functional remission since it cannot be excluded that these limitations regarding the evaluation of the DUP could have an impact on the accuracy of regression estimates and between-group comparisons. Indeed, it is possible that a more precise measurement of DUP with a standardized scale could highlight a cut-off different from that of the 4 weeks demonstrated in our study. The measurement is therefore subject to recall bias and dating uncertainties, particularly in cases of insidious symptom onset. This methodological limitation highlights the need for future studies to use standardized tools for prospective data collection.

Given these potential limitations, future prospective multicentric studies seem needed to validate and expand upon the results of this study.

### 4.5. Clinical Implications and Future Directions

This study reinforces the need to address DUP as a central target for secondary prevention strategies. It also advocates for the early assessment of social determinants of mental health and the systematic integration of psychosocial support services within early intervention programs for FEP [34].

Longitudinal research is necessary to better understand the clinical trajectories following involuntary hospitalization and to identify the most effective therapeutic levers for promoting sustained remission.

Strengthening the coordination between inpatient psychiatry, specialized outpatient care, and early intervention services appears crucial for improving outcomes in young patients with FEP.

## 5. Conclusions

This study highlights the central role of the duration of untreated psychosis (DUP) as an independent predictor of functional non-remission in young adults involuntarily hospitalized for a first episode of psychosis. A DUP exceeding four weeks significantly increases the risk of poor outcomes, even after adjustment for clinical and psychosocial variables [9,20]. These findings support neurobiological hypotheses suggesting that prolonged untreated psychosis may exert lasting neurotoxic effects on brain structures, thereby impairing the potential for remission [22,23,36].

Beyond DUP, contextual factors such as social isolation, low socioeconomic status, unemployment, and lack of structured activity are also strongly associated with functional non-remission [13,14,15,44]. These variables often reflect poor premorbid adjustment and insufficient social support, both of which are key to recovery in FEP.

Although involuntary hospitalization serves as a marker of acute clinical severity, it does not appear to be a direct causal factor for functional non-remission. Rather, it should be interpreted as an indicator of urgent clinical need, warranting a rapid and appropriate therapeutic response [3,8].

Our findings support the expansion of early specialized care services such as ARMS programs, which enable preventive action before the onset of full psychosis [3,24]. A multimodal approach involving optimized pharmacological treatment, psychotherapy, family engagement, and support for social reintegration is essential to improve prognosis [46].

Finally, these results underscore the need to pursue longitudinal and multicentric research to further elucidate post-FEP trajectories and to identify the most effective interventions for preventing chronicity.

## Figures and Tables

**Table 1:** Inclusion and exclusion criteria.

Inclusion Criteria	Exclusion Criteria
Involuntary admission between 1 January 2013 and 31 October 2023	Voluntary admission between 1 January 2013 and 31 October 2023
Age between 18 and 30 years	Age below 18 or above 30 years
No prior psychiatric follow-up for psychosis	Any history of psychotic symptoms prior to the current episode
Presentation of a first episode of psychosis	

**Table 2:** Sociodemographic data (*n* = 123).

Variables	Categories	%	Individuals with Functional Remission	Individuals Without Functional Remission	*p*-Value Chi^2^	OR (CI 95%)	*p*-Value
Sex	Female (*n* = 47)	38.2%	38.3%	38.1%	0.978	1	0.978
	Male (*n* = 76)	61.8%	61.7%	61.9%		1.01 (0.49 to 2.09)	
Age (years)	<25 (*n* = 69)	56.1%	56.7%	55.6%	0.901	1	0.901
	≥25 (*n* = 54)	43.9%	43.3%	44.4%		1.05 (0.51 to 2.13)	
Social Isolation	No (*n* = 68)	55.3%	68.3%	42.9%	0.005	1	0.005
	Yes (*n* = 55)	44.7%	31.7%	57.1%		2.88 (1.38 to 6.02)	
Socioeconomic level of the municipality of residence	Low (*n* = 38)	30.9%	20.0%	41.3%	0.011	1	0.012
	Moderate to high (*n* = 85)	69.1%	80.0%	58.7%		0.36 (0.16 to 0.80)	
Migration history	No (*n* = 30)	24.4%	28.3%	20.6%	0.320	1	0.322
	Yes (*n* = 93)	75.6%	71.7%	79.4%		1.52 (0.66 to 3.48)	
Educational level	Did not complete secondary school (*n* = 38)	30.9%	25.0%	36.5%	0.524	1	0.528
	General secondary education (*n* = 23)	18.7%	18.3%	19.1%		0.71 (0.25 to 2.02)	
	Vocational secondary education (*n* = 33)	26.8%	30.0%	23.8%		0.54 (0.21 to 1.40)	
	Completed higher education (*n* = 29)	23.6%	26.7%	20.6%		0.53 (0.20 to 1.41)	
Professional activity	Job (*n* = 19)	15.5%	21.7%	9.5%	0.028	1	0.033
	Social allowance (*n* = 14)	11.4%	15.0%	7.9%		1.20 (0.28 to 5.18)	
	Studies (*n* = 42)	34.1%	36.7%	31.8%		1.97 (0.63 to 6.17)	
	Unemployment (*n* = 48)	39.0%	26.6%	50.8%		4.33 (1.39 to 13.53)	
Engagement in leisure or sports activities	No (*n* = 70)	56.9%	45.0%	68.3%	0.009	1	0.010
	Yes (*n* = 53)	43.1%	55.0%	31.7%		0.38 (0.18 to 0.79)	
	**Median** **(P25–P75)**				**Wilcoxon test**		
Age (years)	24 (21–27)		23 (20–26)	24 (21–27)	0.428		

**Table 3:** Clinical data on admission (*n* = 123).

Variables	Categories	%	Individuals with Functional Remission	Individuals Without Functional Remission	*p*-Value Chi^2^	OR (CI 95%)	*p*-Value
Family difficulties	No (*n* = 63)	51.2%	56.7%	46.0%	0.238	1	0.239
	Yes (*n* = 60)	48.8%	43.3%	54.0%		1.53 (0.75 to 3.12)	
Previous psychiatric follow-up	No (*n* = 63)	51.2%	46.7%	55.6%	0.324	1	0.325
	Yes (*n* = 60)	48.8%	53.3%	44.4%		0.70 (0.34 to 1.42)	
Previous hospitalizations	No (*n* = 83)	67.5%	65.0%	69.8%	0.567	1	0.567
	Yes (*n* = 40)	32.5%	35.0%	30.2%		0.80 (0.38 to 1.71)	
Previous traumatic events	No (*n* = 77)	62.6%	70.0%	55.6%	0.098	1	0.098
	Yes (*n* = 46)	37.4%	30.0%	44.4%		1.87 (0.89 to 3.92)	
Familial psychiatric historic	No (*n* = 87)	70.7%	71.7%	69.8%	0.824	1	0.824
	Yes (*n* = 36)	29.3%	28.3%	30.2%		1.09 (0.50 to 2.38)	
Duration of psychotic symptoms prior to involuntary hospitalization (weeks)	<4 (*n* = 79)	64.2%	78.3%	50.8%	0.001	1	0.002
	≥4 (*n* = 44)	35.8%	21.7%	49.2%		3.50 (1.59 to 7.70)	
GAF at admission	<30 (*n* = 28)	22.8%	10.0%	34.9%	0.001	1	0.002
	≥30 (*n* = 95)	77.2%	90.0%	65.1%		0.21 (0.08 to 0.56)	
Psychotropic treatment prior to hospitalization	No (*n* = 96)	78.0%	76.7%	79.4%	0.718	1	0.718
	Yes (*n* = 27)	22.0%	23.3%	20.6%		0.85 (0.36 to 2.01)	
Somatic disorder at admission	No (*n* = 98)	79.7%	83.3%	76.2%	0.325	1	0.327
	Yes (*n* = 25)	20.3%	16.7%	23.8%		1.56 (0.64 to 3.82)	
Cannabis use disorder	No (*n* = 55)	44.7%	45.0%	44.4%	0.951	1	0.951
	Yes (*n* = 68)	55.3%	55.0%	55.6%		1.02 (0.50 to 2.08)	
Other substance use disorder	No (*n* = 94)	76.4%	78.3%	74.6%	0.626	1	0.626
	Yes (*n* = 29)	23.6%	21.7%	25.4%		1.23 (0.53 to 2.84)	
Number of psychiatric comorbidities	0 (*n* = 31)	25.2%	30.0%	20.6%	0.377	1	0.381
	1 (*n* = 51)	41.5%	41.7%	41.3%		1.44 (0.59 to 3.54)	
	≥2 (*n* = 41)	33.3%	28.3%	38.1%		1.95 (0.76 to 5.03)	
Suicidal history	No (*n* = 91)	74.0%	73.3%	74.6%	0.873	1	0.873
	Yes (*n* = 32)	26.0%	26.7%	25.4%		0.94 (0.42 to 2.10)	
	**Median** **(P25–P75)**				**Wilcoxon test**		
GAF at admission	35 (30–50)		40 (30–52)	30 (20–40)	0.003		
Duration of psychotic symptoms prior to involuntary hospitalization (weeks)	2 (1–4)		1 (1–3)	3 (1–8)	0.038		

GAF = global assessment of functioning.

**Table 4:** Care-related data (*n* = 123).

Variables	Categories	%	Individuals with Functional Remission	Individuals Without Functional Remission	*p*-Value Chi^2^	OR (CI 95%)	*p*-Value
Duration of hospitalization (weeks)	≤8 (*n* = 104)	84.6%	95.0%	74.6%	0.002	1	0.005
	>8 (*n* = 19)	15.4%	5.0%	25.4%		6.47 (1.78 to 23.55)	
Long-acting injectable antipsychotic	No (*n* = 91)	74.0%	71.7%	76.2%	0.568	1	0.568
	Yes (*n* = 32)	26.0%	28.3%	23.8%		0.79 (0.35 to 1.77)	
Involuntary hospitalization confirmed	Yes (*n* = 75)	61.0%	61.7%	60.3%	0.878	1	0.878
	No (*n* = 48)	39.0%	38.3%	39.7%		1.06 (0.51 to 2.19)	
Prolonged involuntary hospitalization (beyond 40 days)	No (*n* = 88)	71.5%	71.7%	71.4%	0.977	1	0.977
	Yes (*n* = 35)	28.5%	28.3%	28.6%		1.01 (0.46 to 2.22)	
Diagnosis at discharge	Psychotic disorder (*n* = 94)	76.4%	80.0%	73.0%	0.172	1	0.193
	MDD with psychotic symptoms (*n* = 15)	12.2%	6.7%	17.5%		2.87 (0.85 to 9.66)	
	Psychotic mania (*n* = 14)	11.4%	13.3%	9.5%		0.78 (0.25 to 2.43)	
Response	No (*n* = 19)	15.4%					
	Yes (*n* = 104)	84.6%					
Functional remission	No (*n* = 63)	51.2%					
	Yes (*n*=60)	48.8%					
	**Median** **(P25–P75)**				**Wilcoxon test**		
GAF at discharge	65 (60–75)		75 (70–80)	60 (50–61)	<0.001		
Duration of hospitalization (weeks)	6 (2–8)		6 (3–7)	6 (2–9)	0.897		

GAF = global assessment of functioning, MDD = major depressive disorder.

**Table 5:** Multivariate analyses (*n* = 123).

Variable	Model 1 OR Adjusted (CI 95%)	*p*-Value	Model 2 OR Adjusted (CI 95%)	*p*-Value
Duration of psychotic symptoms prior to involuntary hospitalization (weeks)		0.009		0.043
<4	1		1	
≥4	3.11 (1.32 to 7.33)		2.54 (1.03 to 6.24)	
Social Isolation		0.171		0.199
No	1		1	
Yes	1.82 (0.77 to 4.28)		1.82 (0.73 to 4.53)	
Socioeconomic level of the municipality of residence		0.224		0.219
Low	1		1	
Moderate to high	0.56 (0.22 to 1.42)		0.54 (0.20 to 1.44)	
Professional activity		0.335		0.414
Job	1		1	
Social allowance	0.77 (0.15 to 3.98)		0.81 (0.14 to 4.55)	
Studies	1.41 (0.42 to 4.77)		1.30 (0.35 to 4.77)	
Unemployment	2.27 (0.64 to 7.99)		2.20 (0.58 to 8.35)	
Engagement in leisure or sports activities		0.215		0.585
No	1		1	
Yes	0.57 (0.24 to 1.38)		0.77 (0.30 to 1.98)	
GAF at admission				0.083
<30	/		1	
≥30	/		0.37 (0.12 to 1.14)	
Duration of hospitalization (weeks)				0.057
≤8	**/**		1	
>8	/		4.06 (0.96 to 17.17)	

Model 1 = adjusted for social isolation, the socioeconomic level of the municipality of residence, employment status, and sports/leisure activities. Model 2 = adjusted for social isolation, the socioeconomic level of the municipality of residence, employment status, sports/leisure activities, admission GAF score, and length of hospitalization. GAF = Global Assessment of Functioning.

## Data Availability

The data presented in this study are available on request from the corresponding author (the data are not publicly available due to privacy restrictions).
